# Genome evolution following an ecological shift in nectar-dwelling *Acinetobacter*

**DOI:** 10.1128/msphere.01010-24

**Published:** 2024-12-26

**Authors:** Vivianna A. Sanchez, Tanya Renner, Lydia J. Baker, Tory A. Hendry

**Affiliations:** 1 Department of Microbiology, Cornell University 8041 , Ithaca, New York, USA; 2 Department of Entomology, The Pennsylvania State University 8082 , University Park, Pennsylvania, USA; University of Michigan, Ann Arbor, Michigan, USA

**Keywords:** *Acinetobacter*, plant–microbe interactions, evolution, genomics, microbial ecology

## Abstract

The bacterial genus *Acinetobacter* includes species found in environmental habitats like soil and water, as well as taxa adapted to be host-associated or pathogenic. High genetic diversity may allow for this habitat flexibility, but the specific genes underlying switches between habitats are poorly understood. One lineage of *Acinetobacter* has undergone a substantial habitat change by evolving from a presumed soil-dwelling ancestral state to thrive in floral nectar. Here, we compared the genomes of floral-dwelling and pollinator-associated *Acinetobacter*, including newly described species, with genomes from relatives found in other environments to determine the genomic changes associated with this ecological shift. Following one evolutionary origin of floral nectar adaptation, nectar-dwelling *Acinetobacter* taxa have undergone reduction in genome size compared with relatives and have experienced dynamic gene gains and losses as they diversified. Gene content changes suggest a shift to metabolism of monosaccharides rather than diverse carbohydrates, and scavenging of nitrogen sources, which we predict to be beneficial in nectar environments. Gene gains appear to result from duplication events, evolutionary divergence, and horizontal gene transfer. Most notably, nectar-dwelling *Acinetobacter* acquired the ability to degrade pectin from plant pathogens, and the genes underlying this ability have duplicated and are under selection within the clade. We hypothesize that this ability was a key trait for adaptation to floral nectar, as it could improve access to nutrients in the nutritionally unbalanced habitat of nectar. These results identify the genomic changes and traits coinciding with a dramatic habitat switch from soil to floral nectar.

**IMPORTANCE:**

Many bacteria, including the genus *Acinetobacter*, commonly evolve to exploit new habitats. However, the genetic changes that underlie habitat switches are often unknown. Floral nectar is home to specialized microbes that can grow in this nutritionally unbalanced habitat. Several specialized *Acinetobacter* species evolved from soil-dwelling relatives to become common and abundant in floral nectar. Here, we investigate the genomic adaptations required to successfully colonize a novel habitat like floral nectar. We performed comparative genomics analyses between nectar-dwelling *Acinetobacter* and *Acinetobacter* species from other environments, like soil and water. We find that although gene loss coincided with the switch to living in nectar, gains of specific genes from other bacteria may have been particularly important for this ecological change. *Acinetobacter* living in nectar gained genes for degrading pectin, a plant polysaccharide, which may improve access to nutrients in their environment. These findings shed light on how evolutionary novelty evolves in bacteria.

## INTRODUCTION

The gammaproteobacterial genus *Acinetobacter* is diverse and includes taxa that inhabit a broad range of environments, such as soil and water (1). Some *Acinetobacter* lineages have also evolved to be host-associated or animal pathogens, with a notable example being the recently emerged human pathogen *Acinetobacter baumannii* (2). Taxa in the genus are phenotypically and genetically diverse and frequently adapt to new ecological niches (3, 4). However, few direct connections have been made between specific genomic changes and ecological transitions within *Acinetobacter* or in bacteria more broadly (5). One poorly characterized habitat transition within the genus *Acinetobacter* is adaptation for growth in floral nectar. Several *Acinetobacter* species found in floral nectar appear to be most closely related to soil-dwelling relatives (4, 6, 7). Nectar represents a significant environmental shift compared with soil habitats, likely with different selective pressures. Genomic comparisons between *Acinetobacter* adapted to floral nectar versus other habitats could uncover how bacteria evolve to new environments and which genetic traits facilitate major ecological switches.

The high genetic diversity and genomic plasticity within *Acinetobacter* may be driven by mechanisms facilitating horizontal gene transfer (HGT), including competence for natural transformation, conjugative abilities, and mobile elements, such as plasmids, prophage, and insertion sequences (3, 8–11). Horizontally acquired genomic islands are commonly observed throughout *Acinetobacter* (3) and can contain genes conferring beneficial phenotypes like antibiotic resistance (12). HGT is a source of evolutionary novelty in bacteria (13), but other sources of genetic diversity can also be important, such as error-prone DNA polymerases in *A. baumannii* (3, 14), or gene duplication followed by divergence (15). Gene duplication can also potentially lead to increased gene expression, allowing for enhanced nutrient acquisition, temperature stress tolerance, and overall resistance to antibiotics (16), but it is unclear how broadly important this mechanism is for bacterial adaptation.

Genetic novelty in bacteria can allow for the evolution of new traits and subsequent exploitation of new niches (17–20). In some cases, specific genes have been linked to habitat-specific fitness. For instance, *A. baumannii* has antibiotic resistance genes allowing for persistence in hospital settings (2, 21). Antibiotic resistance is a common example of a novel trait resulting from a specific environmental selective pressure because it is easily observable and important in well-studied pathogen systems. In natural systems, specific traits have occasionally been connected to ecological changes in bacteria (22, 23), but such connections can be difficult to infer. In other cases, traits that are linked to success in a specific environment may be known but not their genetic basis. For instance, the ability to access nutrients from pollen is a unique and potentially beneficial trait in floral nectar-dwelling *Acinetobacter*, but how this trait was gained is unknown (24).

Floral nectar is a nutritional reward produced by flowers to attract pollinating animals. It is high in carbohydrates; the sugar content in floral nectar can reach 90% of nectar dry weight (25), and it is a resource for microbes as well (26–31). However, floral nectar habitats create several stresses for microbes, including limitation of nutrients other than sugar (26). Although nectar contains amino acids and lipids (32), it can contain limiting amounts of nitrogen for some microbes (29, 33–35). These factors make nectar a selective environment and can lead to strong priority effects where early arriving microbes prevent subsequent colonization of flowers (33, 36, 37).

Culture-dependent and independent methods have revealed diverse microbes that thrive in these conditions (27, 28, 30, 38–43). The genus *Acinetobacter* makes up a high proportion of bacterial taxa in floral nectar and is prevalent and readily cultured from nectar environments (6, 7, 28, 38, 39, 43). *Acinetobacter* is also frequently found associated with floral visitors. For example, *Acinetobacter apis* was isolated from the gut of the western honey bee, *Apis mellifera*, and bee pollen provisions and nests sometimes include *Acinetobacter* (44–47). However, it is unknown whether *Acinetobacter* found with pollinators are nectar-dwelling species being dispersed by floral visitors, or if they are specific associates of pollinators. For ease here, we refer here to isolates from both nectar and floral visitors as nectar-dwelling.

Previous phylogenomic analysis of *Acinetobacter* isolates from nectar and bees found that they were closely related to soil-dwelling species (7). This work suggested one evolutionary origin of nectar-dwelling/bee association but did not assess evolutionary patterns within this lineage. Here, we study the genome evolution of nectar-dwelling *Acinetobacter* in comparison to taxa isolated from other environments. We include genomes of three previously described species, *A. apis*, *A. boissieri*, and *A. nectaris* (6, 45), newly sequenced *A. nectaris* isolates, and genomes of three recently described species, *A. pollinis*, *A. rathckeae*, and *A. baretiae* (7). For comparison, we included genomes from *A. brisouii*, which is isolated from soil and water and was previously found to be the closest relative of *A. nectaris*, as well as those from eight other environmental *Acinetobacter* species, chosen to represent broad-scale diversity within the genus. We hypothesized that the switch to floral nectar from soil would drastically change the selective pressures experienced by this *Acinetobacter* lineage, leading to changes in gene content. We used comparative genomics to understand which genes may become unnecessary or beneficial for bacteria in floral nectar, and to identify metabolic abilities that may have facilitated this environmental switch.

## RESULTS AND DISCUSSION

### Phylogeny and genome characteristics of nectar-dwelling *Acinetobacter*

**Fig 1:**
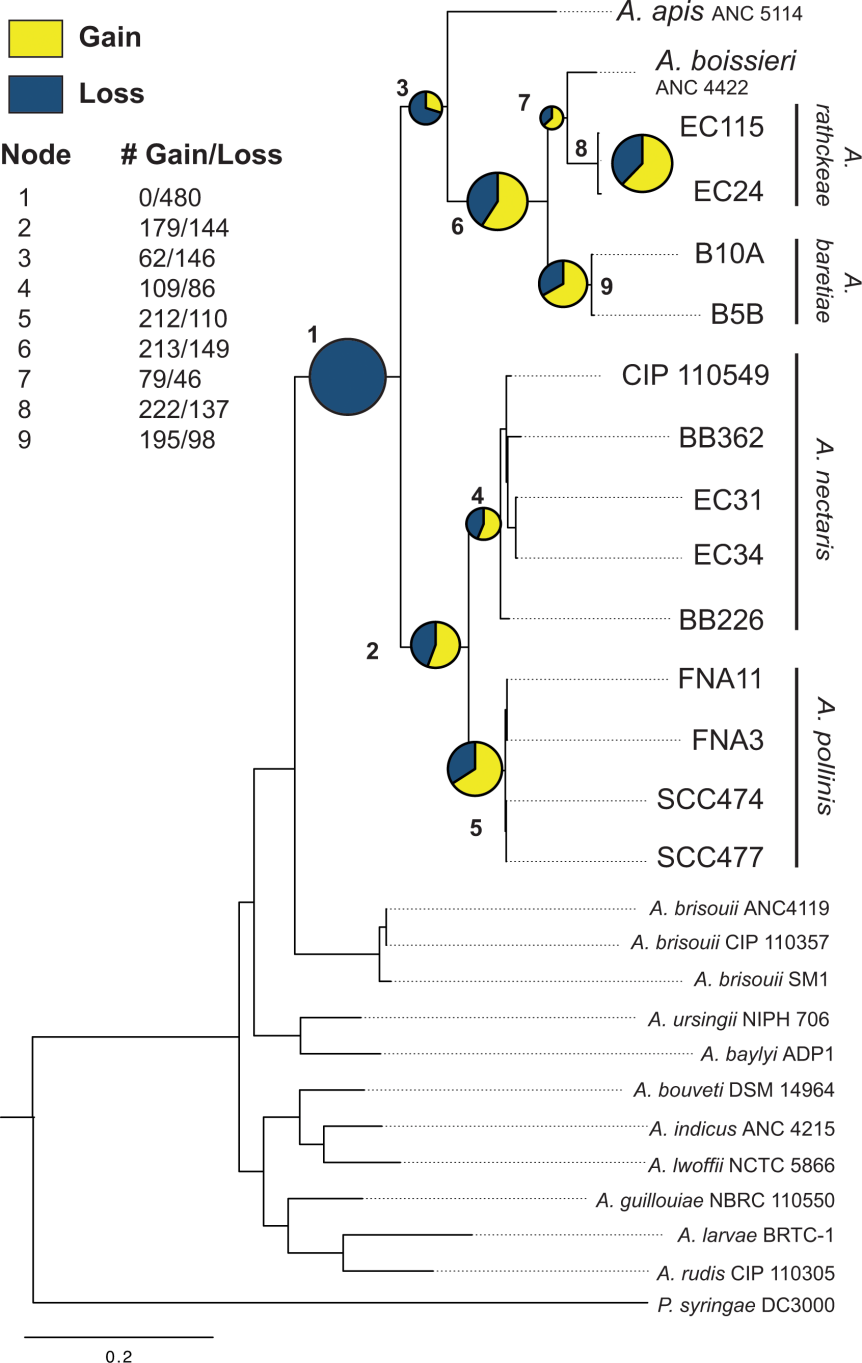
*Acinetobacter* species maximum-likelihood phylogenomic tree based on 399 conserved protein sequences. All nodes have a bootstrap value of 100. Ancestral state reconstruction of ortholog gains and losses at nodes within the nectar-dwelling clade is shown. Nodes are labeled numerically, and a pie chart at each node shows the proportion of ortholog gains (yellow) versus losses (blue). Pie size is scaled by the total number of gain and loss events. Ortholog gains and losses at tips are shown in Fig. **S1**).

To understand the evolutionary history of nectar-dwelling *Acinetobacter*, we constructed a phylogenomic tree using genomes of *Acinetobacter* isolates from floral nectar and floral visitors. The isolates collected from floral nectar and pollinators form a clade, with bootstrap support of 100, separate from soil, water, and animal-dwelling *Acinetobacter* species (Fig. 1). This confirms that there is one known evolutionary origin of nectar-dwelling within *Acinetobacter* and that this group evolved from a presumed soil-dwelling ancestor. The six species in the nectar clade appear to not be isolated from environments outside of floral nectar or pollinators based on 16S rRNA sequence comparisons to GenBank databases (7). Multiple of these species, *A. nectaris*, *A. boissieri*, and *A. pollinis*, are abundant and common in floral nectar from locations worldwide, and our isolates came from both North America and Europe (7). This suggests that members of the clade are specialized for growth in floral nectar and/or associated with pollinators and are widely found in these habitats (7, 28, 38, 41, 43, 45).

We used genomic comparisons between nectar-dwelling *Acinetobacter* and relatives living in distinct environments to uncover genomic patterns associated with nectar specialization. Relative to taxa found in other environments, isolates in the nectar-specialized *Acinetobacter* clade have smaller genomes and lower numbers of protein-coding genes (Table 1). Among all complete reference *Acinetobacter* genomes in GenBank (92 total), there is a significant difference between genome sizes within the nectar-specialist clade, with an average of 2.64 Mb, and the environmental clade, averaging 3.61 Mb (Welch’s *t*-test, one-tailed; df = 17.78, T = −13.10, *P* = <0.000001). Only two non-nectar-dwelling species (out of 86 genomes) had genome sizes that overlapped with those in the nectar clade (Table S1). Across genomes of nectar-dwelling isolates and environmental isolates analyzed here, nectar isolates have 243–977 fewer protein-coding genes, a 10%–30% reduction in proteins.

**TABLE 1:** Genome characteristics of nectar-dwelling *Acinetobacter* genomes and comparison of environmental genomes^*a*^

Isolate	Accession	Complete-ness (%)	Pseudo-genes	Genome size (Mb)	G + C content (%)	Protein-coding genes	Proteins assigned to COGs
*A. apis* ANC 5114^T^	FZLN00000000	100	125	2.41	38.3	2,232	1,985
*A. boissieri* ANC 4422 ^T^	NZ_FMYL00000000	99.9	182	2.69	38.0	2,554	2,378
*A. rathckeae* EC115	VTDP00000000	99.3	192	2.62	39.2	2,438	2,388
*A. rathckeae* EC24	VTDO00000000	99.9	214	2.75	39.3	2,565	2,531
*A. baretiae* B10A ^T^	VTDM00000000	99.9	234	2.59	37.5	2,531	2,402
*A. baretiae* B5B	VTDL00000000	99.5	253	2.70	37.5	2,629	2,486
*A. nectaris* CIP 110549^T^	NZ_AYER00000000	99.3	189	2.67	36.7	2,569	2,458
*A. nectaris* BB362	JAEQDL000000000	96.9	249	2.38	36.9	2,269	2,173
*A. nectaris* EC31	JAERJC000000000	99.9	200	2.42	36.7	2,312	2,303
*A. nectaris* EC34	JAERJB000000000	99.9	212	2.39	36.8	2,288	2,284
*A. nectaris* BB226	JAEQDM000000000	98.7	255	2.58	36.7	2,421	2,312
*A. pollinis* FNA11	VTDS00000000	99.9	245	2.67	36.6	2,621	2,599
*A. pollinis* FNA3	VTDT00000000	99.3	248	2.67	36.6	2,615	2,575
*A. pollinis* SCC474	VTDR00000000	98.1	294	2.75	36.7	2,623	2,605
*A. pollinis* SCC477 ^T^	VTDQ00000000	99.3	262	2.75	36.7	2,634	2,609
*A. brisouii* ANC4119	NZ_APPR00000000	97.7*	187	3.11	41.7	3,036	3,021
*A. brisouii* CIP 110357	NZ_AYEU00000000	97.7*	184	3.09	41.6	3,025	3,003
*A. brisouii* SM1	NZ_JZRE00000000	97.3*	189	2.99	41.8	2,913	2,651
*A. baylyi* ADP1	NC_005966	99.0*	222	3.6	40.4	3,376	2,897
*A. ursingii* NIPH 706	NZ_APQB00000000	99.7*	234	3.49	40	3,468	2,920
*A. bouveti* DSM 14964 ^T^	NZ_APQD00000000	99.7*	198	3.37	40	3,468	2,657
*A. indicus* ANC 4215 ^T^	ATGH01000001	99.9*	238	3.17	45.4	3,064	2,632
*A. lwoffii* NCTC 5866 ^T^	NZ_AYHO00000000	96.0*	268	3.21	43	3,107	2,635
*A. guillouiae* NBRC 110550	NZ_APPJ00000000	99.2*	258	4.88	38.1	4,648	3,362
*A. larvae* BRTC-1 ^T^	NZ_CP016895	97.7*	283	3.74	41.6	3,453	2,598
*A. rudis* CIP110305 ^T^	ATGI00000000	98.9*	241	4	39.1	3,748	2,849

^*a*^ Type strains are labeled, * indicates completeness estimates from GenBank.

Genomic reduction can occur for various reasons. Genome streamlining is common for bacteria living in stable, nutrient-poor conditions, such as some soil and marine habitats (48, 49), and is thought to be driven by selection and facilitated by large effective population sizes (50–52). Additionally, some environmental stresses may promote genome streamlining due to selection (51, 53). Gene loss can also be degenerative and result from genetic drift, with extreme examples occurring in bacteria that are host-restricted and experience frequent population bottlenecks (54, 55). Nectar-dwelling bacteria may experience population bottlenecks due to the transient nature of the floral environment. We therefore tested for evidence of genetic drift as is seen in host-restricted bacteria; such genomes often show high evolutionary rates, high rates of pseudogenes, and low genome GC content (56, 57). Evolutionary rate tests found a higher evolutionary rate for the nectar-dwelling clade (substitution rate relative to environmental taxa = 2.5; −lnL = 54,3761.48) compared with the null hypothesis of a global clock across the nectar and environmental *Acinetobacter* phylogeny (−lnL = 542,853.29; likelihood ratio = 908.19; *P* = <0.000001) (57). Similarly, we found that nectar isolate genomes have slightly lower percent GC compositions relative to genomes of environmental taxa (Table 1). We did not find evidence of genomic degeneration in the form of pseudogenes, as the number of pseudogenes detected in nectar-dwelling *Acinetobacter* ranged from 125 to 294, while environmental *Acinetobacter* had a similar range of 187–283 and other *Acinetobacter* species fall within this range as well (Table 1) (58, 59). We speculate that the reduced genome size in nectar-dwelling *Acinetobacter* could be due to a combination of relaxed selection on some genes after the shift to floral nectar, as well as a relative increase in genetic drift due to population bottlenecks. To further investigate this, we sought to define the gene content and functional capacities of nectar-dwelling species compared with soil-dwelling relatives.

### Gene content evolution with the switch to nectar

To determine the content of predicted proteins among *Acinetobacter* clades, we performed an ortholog clustering analysis to identify shared orthologs, recent paralogs, and unique genes (60) (Table S2). This analysis resulted in 7,334 orthologs in total identified across all genomes, with 1,076 core orthologs present across all genomes. About 40% of total orthologs were only found in environmental isolate genomes, whereas only 16% of orthologs were unique to the nectar-dwelling clade (Table S3), supporting a trend towards gene loss rather than gain in the nectar clade.

To trace gene gain and loss events within the nectar-specialist clade, we performed a maximum-likelihood ancestral state reconstruction analysis. Overall, there have been dynamic gene gains across the evolution of the group. Substantial loss (480 orthologs) occurred at the ancestral node of the nectar-dwelling clade and at the ancestral nodes leading to most nectar-dwelling species (Fig. 1). Gene loss sometimes remained high (98–234 orthologs) even at tips and more recent nodes (Fig. 1; Fig. S1). This pattern is consistent with genome reduction as described above and also suggests that the process of gene loss is still ongoing in nectar-dwelling isolates. Gene gains became higher closer to tips, with gains of ~200 orthologs at some nodes (Fig. 1) and gains as high as 159 orthologs at tips (Fig. S1). Gene gains identified in this analysis, particularly at tips, could be the result of divergence leading to novel orthologs as well as horizontal acquisition of new genes.

**Fig 2:**
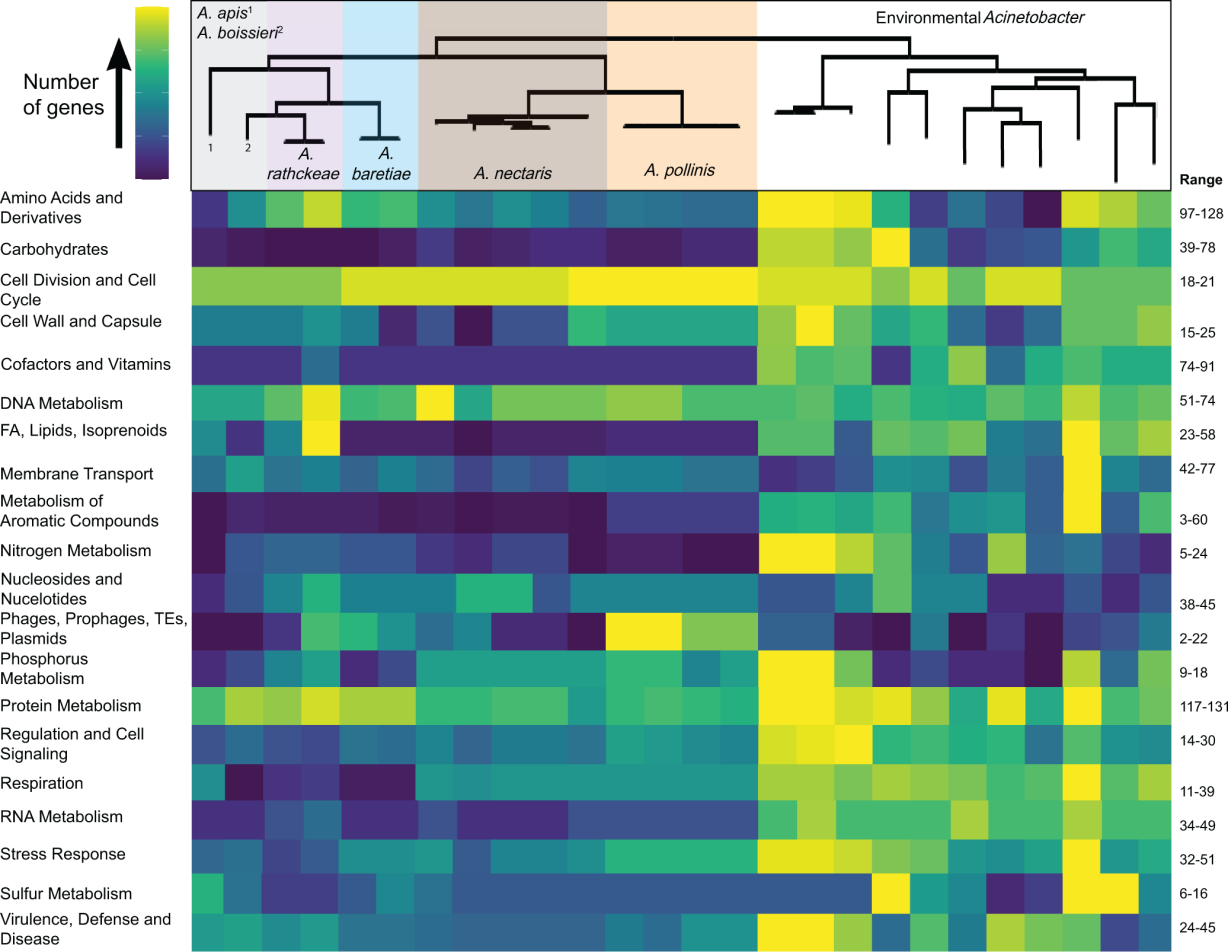
Heatmap of the number of orthologs in each functional category across all genomes. Category assignments are based on RAST annotations (FA = fatty acid metabolism, TE = transposable elements), and the phylogenomic species tree (Fig. 1) shows nectar-dwelling versus environmental isolates. Each row is colored independently based on the variance of ortholog number within the category. The color scale has yellow indicating the highest number of genes and deep blue indicating the lowest number of genes in each row. Genes of unknown function or categories with <6 mean orthologs per genome were excluded, as were uncategorized orthologs and the category of miscellaneous. The range of ortholog numbers across genomes for each category is shown in the right-side column. Specific orthologs are given in Table S2, and statistical analysis of ortholog differences is given in Table S4.

Gene number and content changes in the nectar-dwelling clade compared with environmental relatives occurred across diverse functional categories (Fig. 2). We note that these functions are putative, as they are predicted by homology and categorized by Rapid Annotation using Subsystem Technology (RAST), not confirmed within these taxa. The largest differences were reduced ortholog numbers in nectar-dwelling isolates relative to environmental relatives. Eight (out of 25) functional categories were reduced in nectar-dwelling isolates compared with environmental isolates, ranging from 29% to 77% reduction, and seven functional categories had significantly lower ortholog numbers in the nectar clade (ANOVA analysis, Table S4). Ordered by relative reduction, these significantly reduced categories were metabolism of aromatic compounds, miscellaneous, nitrogen metabolism, fatty acid metabolism, iron acquisition and metabolism, carbohydrate metabolism, and respiration (Fig. 2; Table S4).

We observed a relative increase in ortholog number less often across nectar-dwelling isolates, with only the category of phages and mobile elements and nine subcategories showing significant increases (ANOVA analysis, Table S4). Some categories and subcategories showed high relative increases in ortholog numbers (ranging from 43% increase to more than double), but these typically involved small actual numbers of orthologs (~1–20 mean orthologs per genome), and most subcategories (15 out of 20) were increased by less than 10% in nectar isolates (Table S4). Additionally, three categories showed relatively low reductions (2%–11%) but were significantly increased when the data were normalized by total ortholog number, which we interpret as the category showing less reduction than expected by chance gene loss. These categories were amino acid metabolism, cofactor and vitamin synthesis, and protein metabolism (ANOVA analysis, Table S4). To understand the biological relevance of these differences, we investigated the specific orthologs present in nectar-dwelling versus environmental isolates for functional categories with significant or high (greater than 30%) difference in ortholog numbers (Table S4).

### Metabolism of aromatic compounds

Nectar-dwelling isolate genomes had significantly fewer orthologs predicted to be involved in the metabolism of aromatic compounds (Table S4; Tukey’s HSD test, *P* = <0.000001). With a reduction of 77% compared with environmental isolates, this group showed the highest amount of relative difference of any functional category. The prevalence of specific orthologs in this category was variable across genomes, and nectar-dwelling isolate genomes contained a subset of orthologs that were also found in some environmental genomes (Table S2). Several of the orthologs in nectar isolates are involved in benzoate metabolism, an aromatic compound that is released by plants (61). However, nectar-dwelling *Acinetobacter* genomes were also missing several benzoate metabolism genes present in environmental *Acinetobacter* (Table S2), so there was not a clear functional difference. The decrease in genes in this category suggests that nectar-dwelling *Acinetobacter* may encounter a limited diversity of aromatic compounds compared with species in other environments, but this could also be driven by nectar having less aromatic compound variability than soil or water habitats.

### Nitrogen and amino acid metabolism

Gene content differences in nectar-dwelling isolates compared with environmental relatives suggest that shifts in nitrogen and amino acid metabolism strategies accompanied the switch to nectar dwelling. Nectar-dwelling isolate genomes had 44% fewer orthologs involved in nitrogen metabolism, a significant reduction (Table S4; Tukey’s HSD test, *P* = 0.030). However, we note that this pattern was partly due to an apparent loss of redundancy (Table S2). Orthologs that were missing from nectar isolate genomes, including glutamine and glutamate synthases and ammonium transporters, were present in environmental *Acinetobacter* as several distinct orthologs but were present in nectar-dwelling isolates as only one ortholog.

Since nitrogen metabolism is interconnected with amino acid metabolism, we considered amino acid metabolism ortholog within this context. The category of amino acid metabolism showed a modest (4%) reduction in nectar isolates in untransformed analysis (Table S4) but a significant increase in normalized analysis (Table S4; Tukey’s HSD test, *P* = 0.001), suggesting less loss than expected by chance loss. The subcategories of branched chain amino acids, such as leucine, and histidine metabolism were decreased by 26% and 21%, respectively. We found that genes for degrading leucine and histidine, which were present in greater than 50% of environmental genomes, were absent from all nectar-dweller genomes (Table S2). At the same time, four amino acid metabolism subcategories showed higher ortholog numbers in nectar genomes, including a 71% increase in the subcategory of proline metabolism (Table S4). This difference was mainly due to an increase in genes for the transport of amino acids (Table S2). Overall, we see a loss of amino acid degradation and redundant transporters, as well as gain of additional transporters. Floral nectar is low in nitrogen relative to carbon (29), and the ability to assimilate nitrogen sources has been linked to competition and growth in floral nectar in both yeasts (33) and *Acinetobacter* (34). A shift towards more diverse transport systems for nitrogen sources could be driven by selection for nitrogen scavenging. Additionally, under nitrogen limitation in floral nectar, the use of available amino acids in protein synthesis, rather than their degradation, may be selected for.

### Carbohydrate metabolism

Genes involved in carbohydrate metabolism showed a significant decrease in nectar-dwelling isolates, which had 31% fewer orthologs than environmental isolates (Table S4; Tukey’s HSD test, *P* = 0.002). The pattern was driven by the subcategories of central carbohydrate metabolism and organic acid metabolism (Table S4). Genes for monosaccharide metabolism were more than twice as numerous in nectar isolate genomes (Table S4; Tukey’s HSD test, *P* = 0.00707), although this category contained a small number of orthologs. Compared with environmental habitats, floral nectar consists of simple carbohydrates, including fructose, sucrose, and glucose (25, 62, 63). Many of the nectar-dwelling species can assimilate fructose, and some can assimilate glucose and sucrose (7, 29). In comparison, non-nectar-dwelling species, such as *A. baylyi*, are often unable to utilize fructose, sucrose, or glucose as sole carbon sources, but can metabolize other diverse carbon sources (64, 65).

Consistent with a shift to monosaccharide utilization, we found that phosphotransferase system (PTS) genes specific to nectar sugars are more common in nectar-dwelling isolates compared with environmental isolates (Table 2; Table S2). PTS genes are a common method for bacteria to transport sugars into cells via a phosphorylation cascade (66, 67). PTS can also be involved in sensing and regulation of physiological processes related to sugar, such as carbohydrate active enzymes, chemotaxis, and biofilm formation (67). These multicomponent systems are specific to distinct molecules, including fructose, mannitol, sucrose, and glucose. The sucrose-specific PTS enzyme complex (EIIABC) is present in four out of six nectar-dwelling species but absent from all environmental isolates. Fructose-specific EIIABC complexes were found in all nectar-dwelling isolates and only three environmental taxa. Together, these differences support a shift in carbohydrate usage from those found in soil to monosaccharides present in floral nectar.

**TABLE 2:** Presence and absence of phosphotransferase system (PTS) genes within *Acinetobacter* genomes and the source environment for each isolate (citations provided where available)

Isolate	None	PTS fructose-specific EIIABC complexes	PTS sucrose-specific EIIBC complexes	Environment (source citation)
*A. apis* ANC5114		1	1	Honey bee gut (45)
*A. boissieri* ANC4422		1	1	Floral nectar (6)
*A. rathckeae* EC115		1	1	Floral nectar (7)
*A. rathckeae* EC24		1	1	Floral nectar (7)
*A. baretiae* B10A		1	1	Honey bee gut (7)
*A. baretiae* B5B		1		Honey bee mouth (7)
*A. nectaris* CIP110549		1		Floral nectar (6)
*A. nectaris* BB362		1		Floral nectar (this study)
*A. nectaris* EC31		1		Floral nectar (this study)
*A. nectaris* EC34		1		Floral nectar (this study)
*A. nectaris* BB226		1		Floral nectar (this study)
*A. pollinis* FNA11		1		Floral nectar (7)
*A. pollinis* FNA3		1		Floral nectar (7)
*A. pollinis* SCC474		1		Floral nectar (7)
*A. pollinis* SCC477		1		Floral nectar (7)
*A. brisouii* ANC4119		1		Peat (68)
*A. brisouii* DSM189516 = CIP 110357		1		Unknown
*A. brisouii* ANC 3789 = SM1		1		River in Endau-Rompin National Park
*A. baylyi* CIP107474		1		Activated sludge plant (65)
*A. ursingii* NIPH706	1			Human blood
*A. bouveti* CIP110357	1			Unknown
*A. indicus* ANC4215	1			Unknown
*A. lwoffii* CIP64.10		1		Unknown
*A. guillouiae* NIPH991	1			Human ear swab
*A. larvae* BRTC1	1			Moth larvae (69)
*A. rudis* CIP110305	1			Raw milk and wastewater (70)

We investigated carbohydrate metabolic capabilities in more detail by comparing carbohydrate active enzymes identified using the CAZy database (71, 72). Environmental genomes contained significantly more genes in the glycosyl transferase (GT) families (Fisher’s exact test, *P* = 0.001), with an average of 23 genes per environmental genome and 15 per nectar-dweller genome (Fig. S2). This difference is mainly driven by enzymes in the GT2 and GT4 families. The role of these specific genes within *Acinetobacter* species is unclear, but typically enzymes in these families are involved in the synthesis of cell wall, capsular, and extracellular biofilm polysaccharides (73, 74), suggesting that some of these functions may be different in nectar-dwelling *Acinetobacter*. In support of this, several biofilm formation genes are found in the nectar-dwelling isolates, including *pgaABCD* genes of the poly-β-1,6-*N*-acetyl-D-glucosamine (PGA) operon responsible for the maintenance of biofilm stability (75). Of the 15 nectar-dwelling isolates, nine have the complete PGA operon, while only four out of the 11 environmental isolates have the full operon. *A. pollinis* isolates also have two to six times the number of copies of the *pgaB* gene, which is critical for export of PGA (76), suggesting that biofilm formation, surface attachment, or cell–cell attachment may be important traits in floral nectar or pollinator environments. Additionally, the subcategory of type IV secretion systems contains significantly (31%; Table S4; Tukey’s HSD test, *P* = 0.0111) more orthologs in nectar-dwelling genomes than in environmental genomes. These increased orthologs are all involved in pilin and fimbrial biogenesis (Table S2). For example, the *fimT*/*pilVWXY* gene cluster (77) is absent in environmental genomes and present in most nectar isolate genomes, whereas *pilABCE* and *pilMNOPQ* clusters were present in both groups. This could suggest additional importance of surface attachment for the nectar-dwelling clade.

In contrast to the decrease in GT enzymes, nectar dwellers contain significantly more genes in glycoside hydrolase (GH) families (Fisher’s exact test, *P* = <0.000001), averaging 15 orthologs per genome compared with 13 orthologs per environmental genome (Fig. S2). This pattern was mainly driven by genes in the GH 28 family, which are involved in the breakdown of the polygalacturonic acid backbone of pectin (78). Pectin is a major component of plant cell walls, and we hypothesize, as discussed below, that the ability to degrade this polysaccharide may be beneficial in floral nectar.

### Phage and mobile elements

The number of orthologs in the category of phage/prophage, transposable elements, and plasmids was nearly double in nectar-dwelling isolates compared with environmental isolates (Table S4; Tukey’s HSD test, *P* = 0.023). This increase was driven by a doubled number of orthologs in the subcategory of phages and prophages (Table S4; Tukey’s HSD test, *P* = 0.00041). Considering that environmental *Acinetobacter* are thought to have high numbers of prophage, this increase is notable (3). Consistent with high phage interactions, we also found a significant increase in the number of CRISPR–Cas system orthologs present in nectar isolates (Table S4; ANOVA, *P* = 0.000174). These were genes for Cascade proteins Cas1, Cas3, Csy2, Csy3, and Csy4, present in *A. apis*, *A. rathckeae*, and *A. baretiae* and also present in some environmental isolates, suggesting that CRISPR–Cas systems may be sporadic across *Acinetobacter* from both nectar and the environment (Table S2) (3).

We hypothesized that HGT could be important in conferring novel functions for *Acinetobacter* switching to a new habitat, and so we screened nectar-dwelling genomes for genomic islands. We found genomic islands within all members of the nectar-dwelling clade, and this analysis also identified intact prophages. Gene counts from genomic islands ranged from 111 to 352 genes with approximately 50% annotated as hypothetical and the remainder involved in plasmid or transposon mobilization, phage replication, or Type1 secretion components, suggesting that HGT is facilitated by mobile elements (Table S5).

### Other significant differences

Additional ortholog categories saw significant shifts in nectar-dweller genomes compared with environmental genomes but with unclear connections to function in nectar. For example, iron acquisition and metabolism both showed significant reduction in nectar isolates (37% reduction; Tukey’s HSD test; *P* = 0.000125), although the category had relatively few genes overall (Table S4). Nectar-dwelling isolate genomes were lacking siderophore related orthologs, specifically regulatory (sigma factors) and receptor uptake orthologs. However, siderophore uptake genes were common among significantly increased membrane transport genes present in nectar dwellers but absent in environmental isolates (Table S4; Tukey’s HSD test; *P* = 0.00099), suggesting reliance on different siderophores among nectar versus environmental isolates (Table S2). Additionally, the categories of miscellaneous functions, fatty acid metabolism, and respiration were significantly reduced in nectar isolates (Table S4; Tukey’s HSD test; *P* = 0.011, *P* = 0.0000892, and *P* = 0.002). The fatty acid or respiration genes lost in nectar-dwelling isolates did not provide insight into the biological significance of this change (Table S2). The miscellaneous orthologs absent from nectar-dweller genomes were predicted to catalyze the degradation of aromatic compounds, suggesting that the decrease in this category is related to the observed decrease in metabolism of aromatic compounds.

The categories of cofactor and vitamin synthesis and protein metabolism showed modest decreases in untransformed analyses but significant increases in normalized data (Table S4; Tukey’s HSD test; *P* = 0.001 and *P* = 0.00939). Within the category of cofactors, nectar-dwelling genomes were missing several orthologs involved in folate metabolism (Table S2). At the same time, orthologs involved in pyridoxine metabolism were significantly increased in nectar isolates (Table S4; Tukey’s HSD test; *P* = 0.0439). In the case of protein metabolism, orthologs in protein biosynthesis and degradation were significantly higher in nectar-dwelling genomes (Table S4; Tukey’s HSD test, *P* < 0.000001, *P* = 0.000513). Additionally, the categories of DNA repair (Table S4; Tukey’s HSD test, *P* = 0.00664) and nucleoside and nucleotide metabolism (Table S4; Tukey’s HSD test, *P* = 0.00252) were significantly increased. However, these all showed very low change compared with environmental genomes (3%–7% relative increases), with changes of only 1–4 orthologs per category (Table S4).

### Acquisition and diversification of pectin enzymes

**Fig 3:**
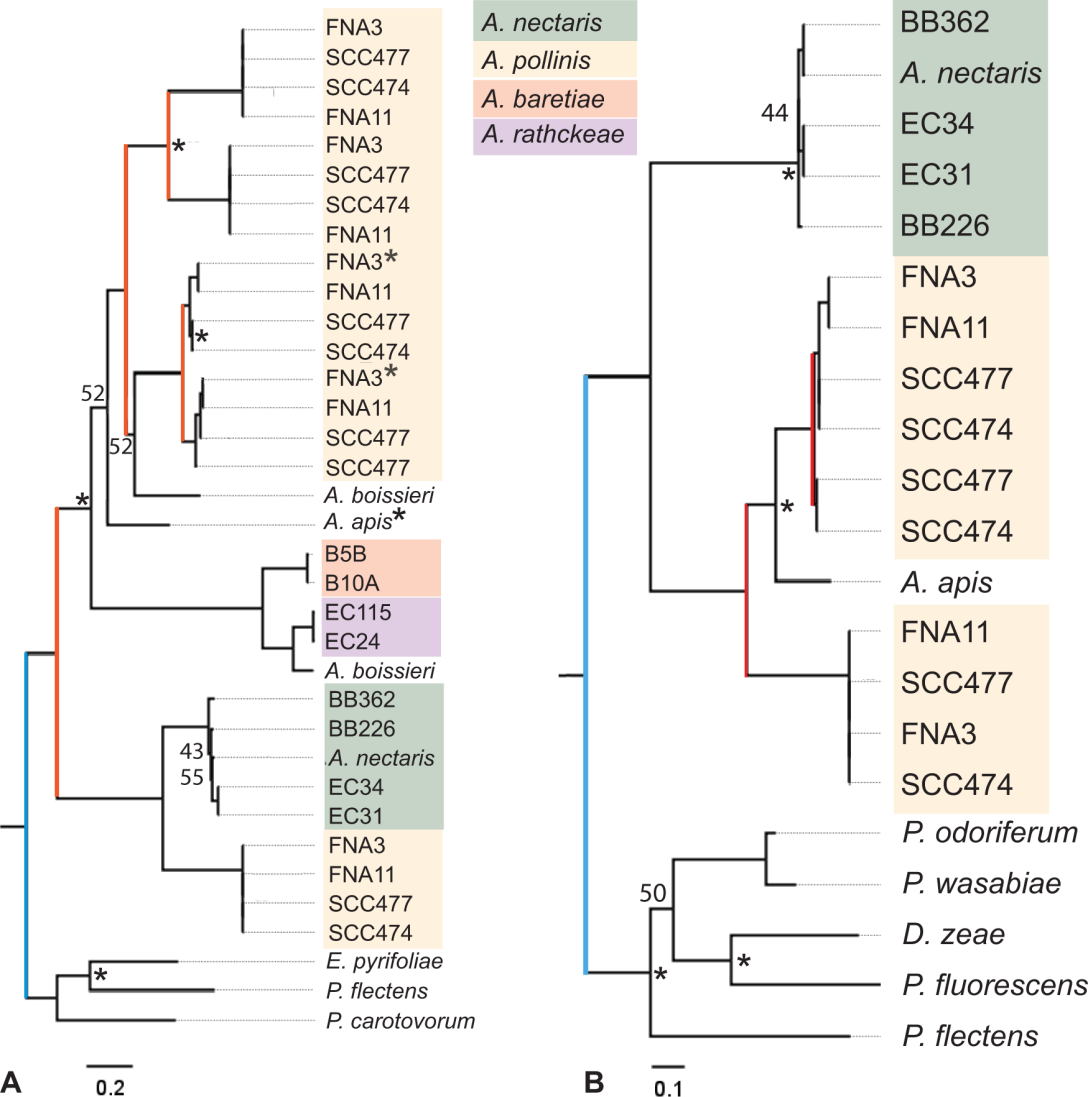
Maximum-likelihood phylogenetic trees of (A) polygalacturonase genes and (B) pectin lyase genes from nectar-dwelling *Acinetobacter* isolates and the most closely related orthologs from plant pathogens (Table S6). Red bars indicate likely duplication events, and blue bars indicate horizontal gene transfer events. Bootstrap values below 80 are displayed at nodes. Asterisks show nodes and tips with significant positive selection. Outgroup orthologs represent the best BLAST hits in GenBank databases.

Among orthologs present in nectar-dwelling isolates and not environmental isolates were genes coding for pectin degradation enzymes, specifically PL1 family pectin lyases and GH28 family polygalacturonases. All species in the nectar-dwelling clade contain at least one of these genes, with several species possessing multiple copies of genes associated with the degradation of pectin (Table S6). Pectin is a recalcitrant polysaccharide that provides structural stability in plant cell walls and the outer layers of pollen grains (79). Among bacteria, enzymes for degrading pectin are commonly found in necrotrophic plant pathogens, which use them to digest plant tissue (78). These enzymes are notably absent among *Acinetobacter* genomes in GenBank, except for orthologs found in the nectar clade. Sequences in GenBank with the highest similarity to nectar-clade orthologs of PL1 and GH28 genes are found outside of *Acinetobacter* in plant pathogens, such as *Pectobacterium*, *Erwinia*, and *Dickeya* (Fig. 3A and B). These genes were likely acquired by nectar-dwelling *Acinetobacter* by HGT, possibly from a necrotrophic plant pathogen in the Enterobacterales.

Tracing the pattern of gains in pectin degradation genes onto the phylogeny of nectar-dwelling *Acinetobacter* suggests that at least one copy each of the pectin lyase and polygalacturonase genes were present in the common ancestor of the nectar-clade *Acinetobacter* analyzed here (Fig. 3A and B). In several isolates, these two orthologs are located next to each other on the chromosome, so they may have been gained together in one event. As seen in the gene trees for the pectin lyase and polygalacturonase orthologs, we determined that these genes experienced multiple duplication events with paralogs sister to each other (Fig. 3A and B). These duplications occurred within the species *A. pollinis*, which contains six copies of polygalacturonase and three copies of pectin lyase (within isolate SCC477 as an example). Additional horizontal transfers, losses, or duplication events may have occurred within the nectar clade, as some species have multiple copies within a gene tree (*A. boissieri*, Fig. 3A) or differences in topology between gene trees and species trees (*A. apis*, Fig. 3B). Some of these copies are on contigs that are likely from plasmids (based on increased read depth and the presence of plasmid replication genes), which may have facilitated duplication and transfers of these genes. Duplication was more common for the polygalacturonase than the pectin lyase genes, and the polygalacturonase genes were also the only example of multiple copies outside of the *A. pollinis* (Fig. 3A).

The fact that these genes have been maintained and duplicated within the nectar-dwelling clade suggests that they may serve an important ecological role for these bacteria. Gene duplication can increase production of protein products (16) but also allows for functional divergence due to selection. To test for this, we performed branch-site tests for positive selection on pectin-degrading genes in nectar-dwelling *Acinetobacter* and unrelated outgroups. Amino acid substitutions in several pectin-degrading enzyme protein sequences show signatures of positive selection (Table S7). Positive selection was detected at the nodes and tips of the polygalacturonase gene tree (Fig. 3A), particularly for *A. pollinis* (nine sites) and *A. apis* (seven sites) orthologs (Table S7). The high number of duplication events of these genes in *A. pollinis*, together with signatures of positive selection, suggests that pectin-degrading enzymes may be functionally diversifying in this species. Both of these enzymes cleave linkages in the polygalacturonic acid backbone of pectin (78). Necrotrophic plant pathogens typically have diverse copies of these enzymes, with slight variations in catalytic ability, to effectively degrade pectin (78, 80, 81). This pattern may be convergently evolving in *A. pollinis*.

**Fig 4:**
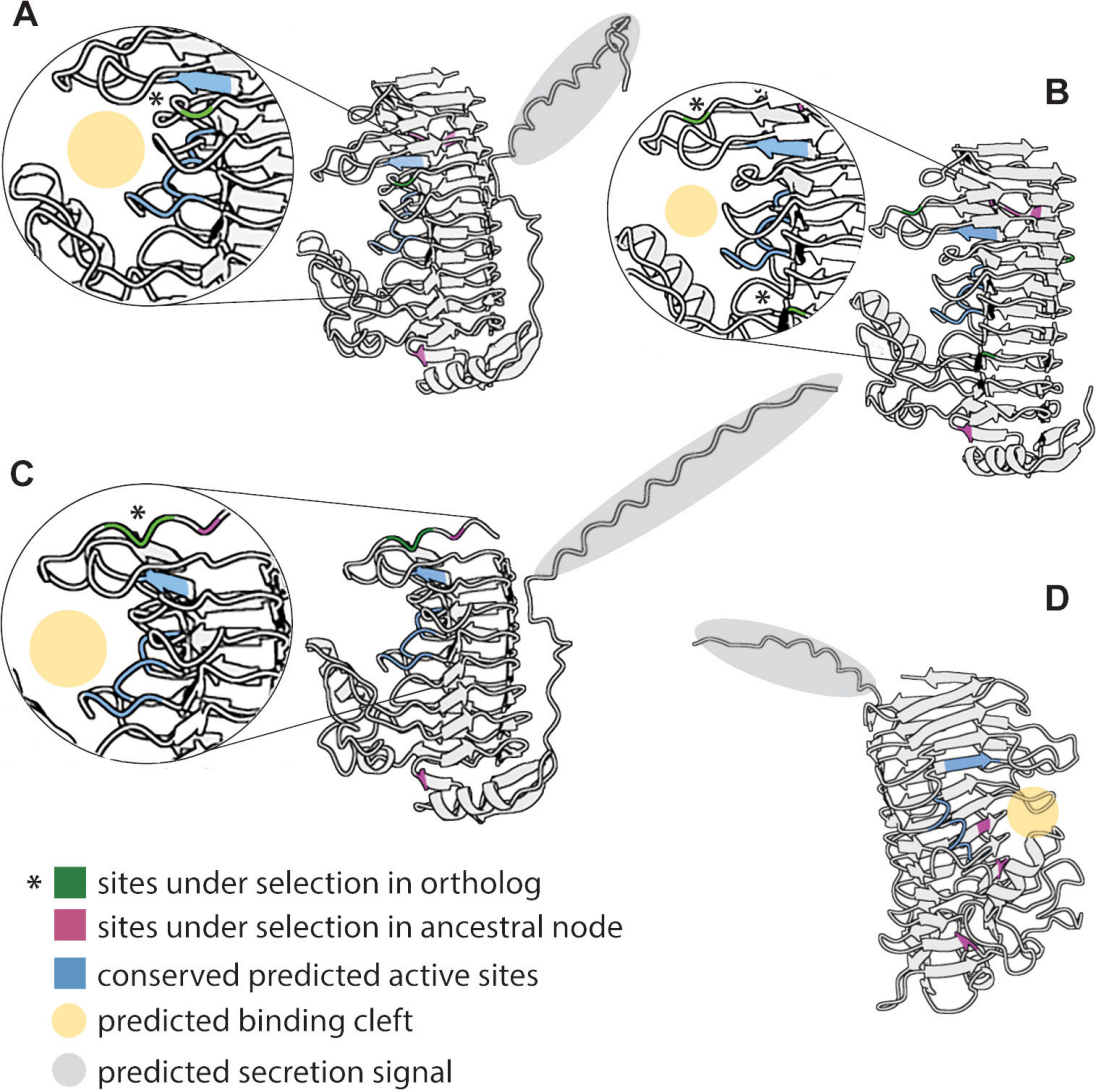
Protein structure predictions of polygalacturonase genes under selection in nectar-dwelling *Acinetobacter*. Shown are representative structures from four clades (Fig. 3) with sites under positive selection detected in either specific orthologs (sites shown in green with an *) or ancestral nodes (sites shown in pink): (**A**) *A. pollinis* FNA3 (locus_tag I2F29_RS12745), (**B**) *A. apis* ANC 5114 (locus_tag CFY84_RS01715), (**C**) *A. pollinis* FNA3 (locus_tag I2F29_RS12925), and (**D**) *A. pollinis* FNA3 (locus_tag I2F29_RS02465). Conserved active site motifs, from published work (82), are highlighted in blue. Secretion signal tags, based on protein domain predictions from Uniprot, are shown in gray, and substrate-binding clefts are shown in yellow (83). Proteins are rotated to best show active sites and sites under selection. For A–C, insets show zoomed in views of the binding cleft and sites under selection.

To investigate the potential function of the amino acids under selection in *Acinetobacter* polygalacturonase enzymes, we generated predicted protein structures of representative orthologs from clades with sites experiencing positive selection at the tips or nodes (Fig. 4). Protein structures were predicted with high confidence and were generally similar to structures of proteins from known plant pathogens (Fig. S3). All nectar isolate proteins included known conserved active motifs, the catalytic sites NTD and RIK, and substrate-binding sites G/QDD and G/SHG (82) in the predicted binding cleft of the enzyme (Fig. 4) (83). Several of the sites found to be under positive selection were also located around the binding cleft. For example, six sites found to be under positive selection in specific orthologs (tips) are predicted to be near the substrate-binding cleft in three orthologs (Fig. 4A through C). Additionally, three sites under selection at ancestral nodes, and therefore present in several orthologs, were also near the binding cleft in two orthologs (Fig. 4C and D). Most of the substitutions found to be under selection (Table S7) were between amino acids that vary in hydrophobicity due to their side chains or charge. Of the nine sites under selection near the binding cleft, five involved substitutions from hydrophilic to hydrophobic amino acids, one involved a substitution in the opposite direction, and three were between similarly hydrophobic amino acids. Previous work found that hydrophilic amino acids in the binding cleft were important for function of polygalacturonase in plant pathogens (83), and our results suggest that changes in hydrophobicity may be beneficial for enzyme activity in floral nectar compared with plant tissue.

It is not known how much nectar-dwelling *Acinetobacter* interact with major sources of pectin in plant tissue, but to our knowledge, they have not been observed to infect plants. However, microbes in nectar regularly interact with pollen grains, which are introduced into nectar by pollinator activity (84). In fact, some *Acinetobacter* can cause pollen grains to burst open or pseudogerminate (24). This ability is beneficial for *Acinetobacter*, as it is associated with increased growth in nectar when pollen is present (24). Floral nectar has been shown to be nitrogen limiting for both yeasts and bacteria (33, 34), so the ability to access nitrogen from pollen in nectar could increase microbial fitness. However, pollen is protected by a resistant exine layer and is difficult to degrade (85). Pectin is an essential component of pollen cell walls and pollen tubes (86), and pectin-degrading enzymes have been hypothesized to be involved in pollen breakdown by bacterial gut symbionts of honey bees (87). We hypothesize that the pectin degradation enzymes in *Acinetobacter* could be involved in accessing nutrients from pollen, which could explain the apparent importance of genes coding for such enzymes in the clade. In support of this, we find that most of the GH28 and PL1 proteins in *Acinetobacter* have secretion signals (Fig. 4A and C through D), similar to secreted pectin-degrading enzymes in *Pectobacterium* (78), suggesting that they should act extracellularly. Furthermore, we find the most selection on these genes within *A. pollinis* and *A. apis*. The former shows strong impacts on pollen bursting and pseudogermination (24), and the latter was isolated from honey bees and is likely to encounter pollen regularly. We speculate that the ability to degrade pectin could be a key trait allowing *Acinetobacter* to thrive in nectar and in association with pollinators.

### Conclusions

We found that the ecological shift from soil-dwelling to nectar-dwelling led to genomic reduction, followed by dynamic gene gains and losses underlying apparent metabolic shifts in diverse functions. The nectar clade had an increase in the number of genes involved in monosaccharide metabolism and transport, likely due to the high sugar environment of nectar. We also found changes in nitrogen and amino acids metabolism genes, suggesting a switch toward nitrogen scavenging relative to environmental *Acinetobacter*, consistent with nitrogen limitation in floral nectar. Nectar-dwelling *Acinetobacter* species have acquired pectin-degrading enzymes, presumably through HGT from plant pathogens. We found duplication, diversification, and positive selection within pectin-degrading genes, supporting our hypothesis that these genes may provide an important ecological function. Overall, we find that genome evolution from gene loss, diversification, and HGT may have all contributed to the *Acinetobacter* habitat switch to floral nectar.

## MATERIALS AND METHODS

### Phylogenetic analyses

A phylogenomic species tree was inferred using 26 *Acinetobacter* genomes and *Pseudomonas syringae* pv. *tomato* strain DC3000 as an outgroup (Table 1). These included sequences from nine environmental *Acinetobacter* species. These genomes were chosen to represent all deep branching clades, with one genome per clade, from the phylogenetic analysis in Garcia-Garcera et al. (4). Based on that analysis, we excluded clades containing the animal pathogens *A. baumannii* or *A. parvus*, as they were found to have undergone rapid and distinct evolutionary changes compared with soil-dwelling relatives (4). We also included a genome of *A. larvae*, which we reasoned might have convergent gene content similarity with pollinator-associated isolated because it originated from a moth larvae gut (69), and all sequenced genomes of *A. brisouii*, which is the closest relative to previously sequenced nectar isolates (4). We included all previously sequenced genomes of *Acinetobacter* isolated from floral nectar or pollinators (6, 7, 45). Additionally, we included newly sequenced *A. nectaris* isolates (EC31, EC34, BB226, and BB362) collected by Rachel Vannette at the University of California, Davis main campus and Bee Biology research facility from the floral nectar of *Epilobium canum*, *Scrophularia californica*, and *Penstemon heteriphyllus*. These isolates were grown using previous methods (7), and DNA was extracted using a Qiagen Blood and Tissue kit and the manufacturer’s instructions. Nextera libraries were prepared using genomic DNA and run on a 2 × 250 paired-end Rapid Run HiSEQ 2500 platform at the Cornell University Institute of Biotechnology Resource Center Genomics Facility. Genomes were assembled using Discovar *de novo* (88) and checked for completeness using the Gammaproteobacteria set of 275 gene markers in CheckM (v1.0.18) (89). Most nectar-dwelling isolate genomes were found to be at least 99% complete (Table 1), with only three with lower completeness scores (96%–98%). We also report genome completeness for comparison environmental isolates (Table 1), which were 96%–99%. These values were taken from GenBank and generated using CheckM with the *Acinetobacter* marker set. All analyzed genomes, both those generated here and those from GenBank, were annotated using the RAST Server for consistency (90). Protein sequences were used in the PhyloPhlAn 3.0 pipeline (91) to determine conserved proteins within *Acinetobacter* genomes. PhyloPhlAn identified 399 conserved proteins and their nucleotide sequences were extracted and concatenated with PhyloPhlAn and aligned using MAFFT (92). Maximum-likelihood trees were reconstructed using IQ Tree (93) with bootstrapping set to 1,000 and a symmetric substitution model. Welch’s *t*-test was used to determine the significance of differences between genome size from the nectar clade and environmental genomes. Tests of evolutionary rate were performed in PAML (94), using the rooted phylogenomic tree to generate likelihood values assuming a global clock (null hypothesis) versus a local clock (alternative hypothesis). The local clock allowed the nectar clade to have a different substitution rate than the rest of the tree. The difference between these likelihoods was tested with a likelihood ratio test in PAML.

Gene trees were reconstructed using polygalacturonase and pectin lyase genes from the *Acinetobacter* genomes. Outgroups were selected using nectar-dwelling *Acinetobacter* spp. pectin lyase and polygalacturonase genes as BLAST queries in GenBank. We found that all of the best BLAST hits for these genes were from necrotrophic plant pathogens, so we included the most similar (>65% identity and >50% query coverage) sequences in the analyses. *Acinetobacter* polygalacturonase and pectin lyase genes were identified in our newly sequenced genomes by RAST annotation, BLAST of the genomes using plant pathogen orthologs as queries, and comparison with the CAZy database to confirm that we had identified all orthologs. Genes were aligned using MAFFT and were used for maximum-likelihood phylogenetic inference in IQ Tree using the TIM3 substitution model, which was selected using model finder.

### Ortholog analyses

Orthologous protein sequence clustering was conducted using OrthoMCL (60) using the following parameters: mode = 1, inflation = 2, pi cutoff = 50. To determine the ancestral state of orthologs across the *Acinetobacter* species tree, the software package Count was used (95), implementing Wagner Parsimony and gene gain penalty of 1.6. This analysis determines the genes gained and lost at each node of the *Acinetobacter* phylogenomic tree minimizing state changes and assuming all character states are reversible, and with the gain penalty gene losses are more likely than gene gains. This ratio of gene loss to gene gain was determined in Count using the rate model optimization tool, with a gain–loss duplication model and a Poisson prior distribution at the root. To estimate the number of pseudogenes present within the genomes, we used the program Pseudofinder (96). The algorithm identifies pseudogenes from GenBank files by analyzing average coding sequence (CDS) length, fragmented CDS, and intergenic pseudogenes, and alignment lengths are compared against homologs identified by blastp hits in the UniProt protein database. The following parameters were used to predict potential intergenic, fragmented, truncated, and long pseudogenes: intergenic length = 30, length pseudo = 0.65, shared hits = 0.5, hitcap = 15, intergenic threshold = 0.3. The webserver tools IslandViewer4 (97) and Phaster (98) were used to identify genomic islands and prophage.

Ortholog enrichment within functional categories for nectar-dwelling versus environmental genomes was performed using ANOVA and Tukey honestly significant difference (HSD) tests using rstatix in R v.4.4.0 and R studio v.2024.4.1.748 (99, 100). Functional category and subcategory classifications were obtained from RAST and only categories with greater than six total orthologs were included in analyses. Uncategorized orthologs were excluded. To account for potential effects of variation in genome size, we analyzed both actual ortholog numbers and numbers normalized by the total ortholog number for each genome. Tukey HSD significance tests were conducted for functional categories and subcategories for both untransformed data and normalized data. Categories and subcategories are presented as consistently reduced or increased (in both untransformed and normalized analyses), as reduced less than expected based on total ortholog number (decreased in untransformed, increased in normalized analyses), or increased less than expected based on total ortholog number (increased in untransformed, decreased in normalized analyses).

### Selection and protein analyses

dN/dS (ω) values were estimated for the polygalacturonase and pectin lyase genes (Table S6) and IQ Tree gene tree maximum-likelihood phylogenies (Fig. 3) using codeml in the PAML v4.4 package, with gaps included (94). Loci with identical sequences between closely related isolates were removed for the analysis. For each tip branch and node in the phylogenies, a likelihood ratio test for positive selection was performed to compare nested branch-site models (Model Anull versus Model A) (Table S7). These analyses allowed for independent comparisons of estimated ω among all branches and subclades (serving as the foreground branches) against the remainder of the phylogeny (background). Tips and nodes were reported as positive for selection if likelihood ratio test results were below the Bonferroni multiple testing correction cut off, and Bayes Empirical Bayes values were above 0.5.

The AI protein prediction software Alphafold was used to predict the structure of pectin degradation enzymes. The Alphafold algorithm is a neural network that generates a multiple sequence alignment from the query protein sequence provided and extracts evolutionary information to generate protein predictions (101). A web version of Alphafold was used for these predictions (102). To determine if amino acid sites under selection were functionally important, we predicted the structure of polygalacturonase orthologs identified to be under positive selection from the PAML analysis, specifically genes from *A. pollinis* isolate FNA3 (GenBank locus tags I2F29_RS12745, I2F29_RS12925, and I2F29_RS11025) and *A. apis* (CFY84_RS01715). Additionally, we compared confidence values for these protein structures to the structure of a protein from a plant pathogen, *Phaseolibacter flectans*. Three-dimensional protein predictions were edited using ChimeraX to highlight sites under selection, as well as predicted active sites (103). Additional domains in the proteins were predicted using the Uniprot database (104).

## Data Availability

All data are available in the supplemental materials or NCBI databases. Genomic assemblies are available in GenBank under accessions VTDP00000000, VTDO00000000, VTDM00000000, VTDL00000000, JAEQDL000000000, JAERJC000000000, JAERJB000000000, JAEQDM000000000, VTDS00000000, VTDT00000000, VTDR00000000, and VTDQ00000000. Sequence reads are available in the SRA under accessions SRR26518995- SRR26519002.

## SUPPLEMENTAL MATERIAL

Supplemental information msphere.01010-24-s0001.docx Supplemental figures and tables.

Table S1 msphere.01010-24-s0002.xlsx Genome sizes of *Acinetobacter* from GenBank.

Table S2 msphere.01010-24-s0003.xlsx All orthologs with RAST functional categories shown.

Table S4 msphere.01010-24-s0004.xlsx ANOVA results across functional categories.

Table S7 msphere.01010-24-s0005.xlsx PAML amino acid selection test results.

ASM does not own the copyrights to Supplemental Material that may be linked to, or accessed through, an article. The authors have granted ASM a non-exclusive, world-wide license to publish the Supplemental Material files. Please contact the corresponding author directly for reuse.

## References

[1] Jung J, Park W. 2015. Acinetobacter species as model microorganisms in environmental microbiology: current state and perspectives. Appl Microbiol Biotechnol 99:2533–2548. doi:10.1007/s00253-015-6439-y 25693672

[2] Peleg AY, Seifert H, Paterson DL. 2008. Acinetobacter baumannii: emergence of a successful pathogen. Clin Microbiol Rev 21:538–582. doi:10.1128/CMR.00058-07 18625687 PMC2493088

[3] Touchon M, Cury J, Yoon E-J, Krizova L, Cerqueira GC, Murphy C, Feldgarden M, Wortman J, Clermont D, Lambert T, Grillot-Courvalin C, Nemec A, Courvalin P, Rocha EPC. 2014. The genomic diversification of the whole Acinetobacter genus: origins, mechanisms, and consequences. Genome Biol Evol 6:2866–2882. doi:10.1093/gbe/evu225 25313016 PMC4224351

[4] Garcia-Garcera M, Touchon M, Brisse S, Rocha EPC. 2017. Metagenomic assessment of the interplay between the environment and the genetic diversification of Acinetobacter. Environ Microbiol 19:5010–5024. doi:10.1111/1462-2920.13949 28967182 PMC5767740

[5] Koskella B, Vos M. 2015. Adaptation in natural microbial populations. Annu Rev Ecol Evol Syst 46:503–522. doi:10.1146/annurev-ecolsys-112414-054458

[6] Álvarez-Pérez S, Lievens B, Jacquemyn H, Herrera CM. 2013. Acinetobacter nectaris sp. nov. and Acinetobacter boissieri sp. nov., isolated from floral nectar of wild Mediterranean insect-pollinated plants. Int J Syst Evol Microbiol 63:1532–1539. doi:10.1099/ijs.0.043489-0 22904213

[7] Alvarez Perez S, Baker LJ, Morris MM, Tsuji K, Sanchez VA, Fukami T, Vannette RL, Lievens B, Hendry TA. 2021. Acinetobacter pollinis sp. nov., Acinetobacter baretiae sp. nov. and Acinetobacter rathckeae sp. nov., isolated from floral nectar and honey bees. Int J Syst Evol Microbiol 71:004783. doi:10.1099/ijsem.0.004783 33970854

[8] Oliveira PH, Touchon M, Cury J, Rocha EPC. 2017. The chromosomal organization of horizontal gene transfer in bacteria. Nat Commun 8:841. doi:10.1038/s41467-017-00808-w 29018197 PMC5635113

[9] Correa A, Shehreen S, Machado LC, Thesier J, Cunic LM, Petassi MT, Chu J, Kapili BJ, Jia Y, England KA, Peters JE. 2024. Novel mechanisms of diversity generation in Acinetobacter baumannii resistance islands driven by Tn7-like elements. Nucleic Acids Res 52:3180–3198. doi:10.1093/nar/gkae129 38407477 PMC11014353

[10] Fondi M, Bacci G, Brilli M, Papaleo MC, Mengoni A, Vaneechoutte M, Dijkshoorn L, Fani R. 2010. Exploring the evolutionary dynamics of plasmids: the Acinetobacter pan-plasmidome. BMC Evol Biol 10:59. doi:10.1186/1471-2148-10-59 20181243 PMC2848654

[11] Traglia GM, Chua K, Centrón D, Tolmasky ME, Ramírez MS. 2014. Whole-genome sequence analysis of the naturally competent Acinetobacter baumannii clinical isolate A118. Genome Biol Evol 6:2235–2239. doi:10.1093/gbe/evu176 25164683 PMC4202317

[12] Sezmis AL, Woods LC, Peleg AY, McDonald MJ. 2023. Horizontal gene transfer, fitness costs and mobility shape the spread of antibiotic resistance genes into experimental populations of Acinetobacter baylyi. Mol Biol Evol 40:msad028. doi:10.1093/molbev/msad028 36788632 PMC9985319

[13] Treangen TJ, Rocha EPC. 2011. Horizontal transfer, not duplication, drives the expansion of protein families in prokaryotes. PLOS Genet 7:e1001284. doi:10.1371/journal.pgen.1001284 21298028 PMC3029252

[14] Norton MD, Spilkia AJ, Godoy VG. 2013. Antibiotic resistance acquired through a DNA damage-inducible response in Acinetobacter baumannii. J Bacteriol 195:1335–1345. doi:10.1128/JB.02176-12 23316046 PMC3591989

[15] Kassen R. 2019. Experimental evolution of innovation and novelty. Trends Ecol Evol 34:712–722. doi:10.1016/j.tree.2019.03.008 31027838 PMC6642843

[16] Kondrashov FA. 2012. Gene duplication as a mechanism of genomic adaptation to a changing environment. Proc Biol Sci 279:5048–5057. doi:10.1098/rspb.2012.1108 22977152 PMC3497230

[17] Colombi E, Hill Y, Lines R, Sullivan JT, Kohlmeier MG, Christophersen CT, Ronson CW, Terpolilli JJ, Ramsay JP. 2023. Population genomics of Australian indigenous Mesorhizobium reveals diverse nonsymbiotic genospecies capable of nitrogen-fixing symbioses following horizontal gene transfer. Microb Genom 9:mgen000918. doi:10.1099/mgen.0.000918 36748564 PMC9973854

[18] Ochman H, Lawrence JG, Groisman EA. 2000. Lateral gene transfer and the nature of bacterial innovation. Nature 405:299–304. doi:10.1038/35012500 10830951

[19] Arnold BJ, Huang I-T, Hanage WP. 2022. Horizontal gene transfer and adaptive evolution in bacteria. Nat Rev Microbiol 20:206–218. doi:10.1038/s41579-021-00650-4 34773098

[20] Bolhuis H, Severin I, Confurius-Guns V, Wollenzien UIA, Stal LJ. 2010. Horizontal transfer of the nitrogen fixation gene cluster in the cyanobacterium Microcoleus chthonoplastes. ISME J 4:121–130. doi:10.1038/ismej.2009.99 19741736

[21] Fournier P-E, Vallenet D, Barbe V, Audic S, Ogata H, Poirel L, Richet H, Robert C, Mangenot S, Abergel C, Nordmann P, Weissenbach J, Raoult D, Claverie J-M. 2006. Comparative genomics of multidrug resistance in Acinetobacter baumannii. PLoS Genet 2:e7. doi:10.1371/journal.pgen.0020007 16415984 PMC1326220

[22] Savory EA, Fuller SL, Weisberg AJ, Thomas WJ, Gordon MI, Stevens DM, Creason AL, Belcher MS, Serdani M, Wiseman MS, Grünwald NJ, Putnam ML, Chang JH. 2017. Evolutionary transitions between beneficial and phytopathogenic Rhodococcus challenge disease management. Elife 6:e30925. doi:10.7554/eLife.30925 29231813 PMC5726852

[23] Melnyk RA, Hossain SS, Haney CH. 2019. Convergent gain and loss of genomic islands drive lifestyle changes in plant-associated Pseudomonas. ISME J 13:1575–1588. doi:10.1038/s41396-019-0372-5 30787396 PMC6776051

[24] Christensen SM, Munkres I, Vannette RL. 2021. Nectar bacteria stimulate pollen germination and bursting to enhance microbial fitness. Curr Biol 31:4373–4380. doi:10.1016/j.cub.2021.07.016 34324834

[25] Pamminger T, Becker R, Himmelreich S, Schneider CW, Bergtold M. 2019. The nectar report: quantitative review of nectar sugar concentrations offered by bee visited flowers in agricultural and non-agricultural landscapes. PeerJ 7:e6329. doi:10.7717/peerj.6329 30834180 PMC6397631

[26] Vannette RL. 2020. The floral microbiome: plant, pollinator, and microbial perspectives. Annu Rev Ecol Evol Syst 51:363–386. doi:10.1146/annurev-ecolsys-011720-013401

[27] Alvarez Perez S, Herrera CM, de Vega C. 2012. Zooming-in on floral nectar: a first exploration of nectar-associated bacteria in wild plant communities. FEMS Microbiol Ecol 80:591–602. doi:10.1111/j.1574-6941.2012.01329.x 22324904

[28] Alvarez Perez S, Herrera CM. 2013. Composition, richness and nonrandom assembly of culturable bacterial-microfungal communities in floral nectar of Mediterranean plants. FEMS Microbiol Ecol 83:685–699. doi:10.1111/1574-6941.12027 23057414

[29] Morales-Poole JR, de Vega C, Tsuji K, Jacquemyn H, Junker RR, Herrera CM, Michiels C, Lievens B, Álvarez-Pérez S. 2023. Sugar concentration, nitrogen availability, and phylogenetic factors determine the ability of Acinetobacter spp. and Rosenbergiella spp. to grow in floral nectar. Microb Ecol 86:377–391. doi:10.1007/s00248-022-02088-4 35930073 PMC10293439

[30] Morris MM, Frixione NJ, Burkert AC, Dinsdale EA, Vannette RL. 2020. Microbial abundance, composition, and function in nectar are shaped by flower visitor identity. FEMS Microbiol Ecol 96:fiaa003. doi:10.1093/femsec/fiaa003 31922546

[31] Parachnowitsch AL, Manson JS, Sletvold N. 2019. Evolutionary ecology of nectar. Ann Bot 123:247–261. doi:10.1093/aob/mcy132 30032269 PMC6344224

[32] Nepi M. 2014. Beyond nectar sweetness: the hidden ecological role of non‐protein amino acids in nectar. J Ecol 102:108–115. doi:10.1111/1365-2745.12170

[33] Dhami MK, Hartwig T, Fukami T. 2016. Genetic basis of priority effects: insights from nectar yeast. Proc Biol Sci 283:20161455. doi:10.1098/rspb.2016.1455 27708148 PMC5069511

[34] Álvarez-Pérez S, Tsuji K, Donald M, Van Assche A, Vannette RL, Herrera CM, Jacquemyn H, Fukami T, Lievens B. 2021. Nitrogen assimilation varies among clades of nectar- and insect-associated Acinetobacters. Microb Ecol 81:990–1003. doi:10.1007/s00248-020-01671-x 33404822

[35] Pozo MI, Jacquemyn H. 2019. Addition of pollen increases growth of nectar-living yeasts. FEMS Microbiol Lett 366:fnz191. doi:10.1093/femsle/fnz191 31550375

[36] Chappell CR, Dhami MK, Bitter MC, Czech L, Herrera Paredes S, Barrie FB, Calderón Y, Eritano K, Golden L-A, Hekmat-Scafe D, Hsu V, Kieschnick C, Malladi S, Rush N, Fukami T. 2022. Wide-ranging consequences of priority effects governed by an overarching factor. Elife 11:e79647. doi:10.7554/eLife.79647 36300797 PMC9671501

[37] Tucker CM, Fukami T. 2014. Environmental variability counteracts priority effects to facilitate species coexistence: evidence from nectar microbes. Proc Biol Sci 281:20132637. doi:10.1098/rspb.2013.2637 24430846 PMC3906935

[38] Fridman S, Izhaki I, Gerchman Y, Halpern M. 2012. Bacterial communities in floral nectar. Environ Microbiol Rep 4:97–104. doi:10.1111/j.1758-2229.2011.00309.x 23757235

[39] Aizenberg-Gershtein Y, Izhaki I, Halpern M. 2013. Do honeybees shape the bacterial community composition in floral nectar? PLoS ONE 8:e67556. doi:10.1371/journal.pone.0067556 23844027 PMC3701072

[40] Samuni-Blank M, Izhaki I, Laviad S, Bar-Massada A, Gerchman Y, Halpern M. 2014. The role of abiotic environmental conditions and herbivory in shaping bacterial community composition in floral nectar. PLoS ONE 9:e99107. doi:10.1371/journal.pone.0099107 24922317 PMC4055640

[41] von Arx M, Moore A, Davidowitz G, Arnold AE. 2019. Diversity and distribution of microbial communities in floral nectar of two night-blooming plants of the Sonoran Desert. PLoS ONE 14:e0225309. doi:10.1371/journal.pone.0225309 31830071 PMC6907802

[42] Herrera CM, de Vega C, Canto A, Pozo MI. 2009. Yeasts in floral nectar: a quantitative survey. Ann Bot 103:1415–1423. doi:10.1093/aob/mcp026 19208669 PMC2701759

[43] Russell KA, McFrederick QS. 2022. Elevated temperature may affect nectar microbes, nectar sugars, and bumble bee foraging preference. Microb Ecol 84:473–482. doi:10.1007/s00248-021-01881-x 34596711 PMC9436853

[44] Graystock P, Rehan SM, McFrederick QS. 2017. Hunting for healthy microbiomes: determining the core microbiomes of Ceratina, Megalopta, and Apis bees and how they associate with microbes in bee collected pollen. Conserv Genet 18:701–711. doi:10.1007/s10592-017-0937-7

[45] Kim PS, Shin N-R, Kim JY, Yun J-H, Hyun D-W, Bae J-W. 2014. Acinetobacter apis sp. nov., isolated from the intestinal tract of a honey bee, Apis mellifera. J Microbiol 52:639–645. doi:10.1007/s12275-014-4078-0 25098562

[46] D Evans J, Armstrong T-N. 2005. Inhibition of the American foulbrood bacterium, Paenibacillus larvae larvae, by bacteria isolated from honey bees. J Apic Res 44:168–171. doi:10.1080/00218839.2005.11101173

[47] Sarton-Lohéac G, Nunes da Silva CG, Mazel F, Baud G, de Bakker V, Das S, El Chazli Y, Ellegaard K, Garcia-Garcera M, Glover N, Liberti J, Nacif Marçal L, Prasad A, Somerville V, Bonilla-Rosso G, Engel P, SAGE class 2019-2020 and 2020-2021. 2023. Deep divergence and genomic diversification of gut symbionts of neotropical stingless bees. MBio 14:e0353822. doi:10.1128/mbio.03538-22 36939321 PMC10128065

[48] Giovannoni SJ, Cameron Thrash J, Temperton B. 2014. Implications of streamlining theory for microbial ecology. ISME J 8:1553–1565. doi:10.1038/ismej.2014.60 24739623 PMC4817614

[49] Giovannoni SJ, Tripp HJ, Givan S, Podar M, Vergin KL, Baptista D, Bibbs L, Eads J, Richardson TH, Noordewier M, Rappé MS, Short JM, Carrington JC, Mathur EJ. 2005. Genome streamlining in a cosmopolitan oceanic bacterium. Science 309:1242–1245. doi:10.1126/science.1114057 16109880

[50] Martinez-Gutierrez CA, Aylward FO. 2019. Strong purifying selection is associated with genome streamlining in epipelagic Marinimicrobia. Genome Biol Evol 11:2887–2894. doi:10.1093/gbe/evz201 31539038 PMC6798728

[51] Sabath N, Ferrada E, Barve A, Wagner A. 2013. Growth temperature and genome size in bacteria are negatively correlated, suggesting genomic streamlining during thermal adaptation. Genome Biol Evol 5:966–977. doi:10.1093/gbe/evt050 23563968 PMC3673621

[52] Luo H, Friedman R, Tang J, Hughes AL. 2011. Genome reduction by deletion of paralogs in the marine cyanobacterium Prochlorococcus. Mol Biol Evol 28:2751–2760. doi:10.1093/molbev/msr081 21531921 PMC3203624

[53] Simonsen AK. 2022. Environmental stress leads to genome streamlining in a widely distributed species of soil bacteria. ISME J 16:423–434. doi:10.1038/s41396-021-01082-x 34408268 PMC8776746

[54] Moran NA. 1996. Accelerated evolution and Muller’s rachet in endosymbiotic bacteria. Proc Natl Acad Sci U S A 93:2873–2878. doi:10.1073/pnas.93.7.2873 8610134 PMC39726

[55] McCutcheon JP, Moran NA. 2012. Extreme genome reduction in symbiotic bacteria. Nat Rev Microbiol 10:13–26. doi:10.1038/nrmicro2670 22064560

[56] Hendry TA, de Wet JR, Dunlap PV. 2014. Genomic signatures of obligate host dependence in the luminous bacterial symbiont of a vertebrate. Environ Microbiol 16:2611–2622. doi:10.1111/1462-2920.12302 24118864

[57] Hendry TA, Freed LL, Fader D, Fenolio D, Sutton TT, Lopez JV. 2018. Ongoing transposon-mediated genome reduction in the luminous bacterial symbionts of deep-sea ceratioid anglerfishes. MBio 9:e01033-18. doi:10.1128/mBio.01033-18 29946051 PMC6020299

[58] Repizo GD, Espariz M, Seravalle JL, Díaz Miloslavich JI, Steimbrüch BA, Shuman HA, Viale AM. 2020. Acinetobacter baumannii NCIMB8209: a rare environmental strain displaying extensive insertion sequence-mediated genome remodeling resulting in the loss of exposed cell structures and defensive mechanisms. mSphere 5:e00404. doi:10.1128/msphere.00404-20 32727858 PMC7392541

[59] Vallenet D, Nordmann P, Barbe V, Poirel L, Mangenot S, Bataille E, Dossat C, Gas S, Kreimeyer A, Lenoble P, Oztas S, Poulain J, Segurens B, Robert C, Abergel C, Claverie J-M, Raoult D, Médigue C, Weissenbach J, Cruveiller S. 2008. Comparative analysis of Acinetobacters: three genomes for three lifestyles. PLoS One 3:e1805. doi:10.1371/journal.pone.0001805 18350144 PMC2265553

[60] Li L, Stoeckert CJ, Roos DS. 2003. OrthoMCL: identification of ortholog groups for eukaryotic genomes. Genome Res 13:2178–2189. doi:10.1101/gr.1224503 12952885 PMC403725

[61] Widhalm JR, Dudareva N. 2015. A familiar ring to it: biosynthesis of plant benzoic acids. Mol Plant 8:83–97. doi:10.1016/j.molp.2014.12.001 25578274

[62] Chalcoff VR, Aizen MA, Galetto L. 2006. Nectar concentration and composition of 26 species from the temperate forest of South America. Ann Bot 97:413–421. doi:10.1093/aob/mcj043 16373370 PMC2803636

[63] Wolff D. 2006. Nectar sugar composition and volumes of 47 species of Gentianales from a southern Ecuadorian montane forest. Ann Bot 97:767–777. doi:10.1093/aob/mcl033 16495315 PMC2803417

[64] Durot M, Le Fèvre F, de Berardinis V, Kreimeyer A, Vallenet D, Combe C, Smidtas S, Salanoubat M, Weissenbach J, Schachter V. 2008. Iterative reconstruction of a global metabolic model of Acinetobacter baylyi ADP1 using high-throughput growth phenotype and gene essentiality data. BMC Syst Biol 2:85. doi:10.1186/1752-0509-2-85 18840283 PMC2606687

[65] Salcedo-Vite K, Sigala J-C, Segura D, Gosset G, Martinez A. 2019. Acinetobacter baylyi ADP1 growth performance and lipid accumulation on different carbon sources. Appl Microbiol Biotechnol 103:6217–6229. doi:10.1007/s00253-019-09910-z 31144015

[66] Lengeler JW, Jahreis K. 2009. Bacterial PEP-dependent carbohydrate: phosphotransferase systems couple sensing and global control mechanisms. Contrib Microbiol 16:65–87. doi:10.1159/000219373 19494579

[67] Saier MH. 2015. The bacterial phosphotransferase system: new frontiers 50 years after its discovery. J Mol Microbiol Biotechnol 25:73–78. doi:10.1159/000381215 26159069 PMC4512285

[68] Anandham R, Weon H-Y, Kim S-J, Kim Y-S, Kim B-Y, Kwon S-W. 2010. Acinetobacter brisouii sp. nov., isolated from a wetland in Korea. J Microbiol 48:36–39. doi:10.1007/s12275-009-0132-8 20221727

[69] Liu S, Wang Y, Ruan Z, Ma K, Wu B, Xu Y, Wang J, You Y, He M, Hu G. 2017. Acinetobacter larvae sp. nov., isolated from the larval gut of Omphisa fuscidentalis. Int J Syst Evol Microbiol 67:806–811. doi:10.1099/ijsem.0.001644 27902257

[70] Vaz-Moreira I, Novo A, Hantsis-Zacharov E, Lopes AR, Gomila M, Nunes OC, Manaia CM, Halpern M. 2011. Acinetobacter rudis sp. nov., isolated from raw milk and raw wastewater. Int J Syst Evol Microbiol 61:2837–2843. doi:10.1099/ijs.0.027045-0 21239566

[71] Yin Y, Mao X, Yang J, Chen X, Mao F, Xu Y. 2012. dbCAN: a web resource for automated carbohydrate-active enzyme annotation. Nucleic Acids Res 40:W445–51. doi:10.1093/nar/gks479 22645317 PMC3394287

[72] Drula E, Garron M-L, Dogan S, Lombard V, Henrissat B, Terrapon N. 2022. The carbohydrate-active enzyme database: functions and literature. Nucleic Acids Res 50:D571–D577. doi:10.1093/nar/gkab1045 34850161 PMC8728194

[73] Coutinho PM, Deleury E, Davies GJ, Henrissat B. 2003. An evolving hierarchical family classification for glycosyltransferases. J Mol Biol 328:307–317. doi:10.1016/s0022-2836(03)00307-3 12691742

[74] Harding CM, Hennon SW, Feldman MF. 2018. Uncovering the mechanisms of Acinetobacter baumannii virulence. Nat Rev Microbiol 16:91–102. doi:10.1038/nrmicro.2017.148 29249812 PMC6571207

[75] Itoh Y, Rice JD, Goller C, Pannuri A, Taylor J, Meisner J, Beveridge TJ, Preston JF, Romeo T. 2008. Roles of pgaABCD genes in synthesis, modification, and export of the Escherichia coli biofilm adhesin poly-beta-1,6-N-acetyl-D-glucosamine. J Bacteriol 190:3670–3680. doi:10.1128/JB.01920-07 18359807 PMC2394981

[76] Little DJ, Pfoh R, Le Mauff F, Bamford NC, Notte C, Baker P, Guragain M, Robinson H, Pier GB, Nitz M, Deora R, Sheppard DC, Howell PL. 2018. PgaB orthologues contain a glycoside hydrolase domain that cleaves deacetylated poly-β(1,6)-N-acetylglucosamine and can disrupt bacterial biofilms. PLoS Pathog 14:e1006998. doi:10.1371/journal.ppat.1006998 29684093 PMC5933820

[77] Bhattacharyya A, Banerjee G, Chattopadhyay P. 2024. Probable role of type IV pili of Aeromonas hydrophila in human pathogenicity. Pathogens 13:365. doi:10.3390/pathogens13050365 38787217 PMC11124393

[78] Abbott DW, Boraston AB. 2008. Structural biology of pectin degradation by Enterobacteriaceae. Microbiol Mol Biol Rev 72:301–316, doi:10.1128/MMBR.00038-07 18535148 PMC2415742

[79] Cankar K, Kortstee A, Toonen MAJ, Wolters-Arts M, Houbein R, Mariani C, Ulvskov P, Jorgensen B, Schols HA, Visser RGF, Trindade LM. 2014. Pectic arabinan side chains are essential for pollen cell wall integrity during pollen development. Plant Biotechnol J 12:492–502. doi:10.1111/pbi.12156 24428422

[80] Sprockett DD, Piontkivska H, Blackwood CB. 2011. Evolutionary analysis of glycosyl hydrolase family 28 (GH28) suggests lineage-specific expansions in necrotrophic fungal pathogens. Gene 479:29–36. doi:10.1016/j.gene.2011.02.009 21354463

[81] Hugouvieux-Cotte-Pattat N, Condemine G, Shevchik VE. 2014. Bacterial pectate lyases, structural and functional diversity. Environ Microbiol Rep 6:427–440. doi:10.1111/1758-2229.12166 25646533

[82] Palanivelu P. 2006. Polygalacturonase: active site analyses and mechanism of action. Indian J Biotechnol 5:148–162.

[83] van Santen Y, Benen JA, Schröter KH, Kalk KH, Armand S, Visser J, Dijkstra BW. 1999. 1.68-Å crystal structure of endopolygalacturonase II from Aspergillus niger and identification of active site residues by site-directed mutagenesis. J Biol Chem 274:30474–30480. doi:10.1074/jbc.274.43.30474 10521427

[84] Herrera CM. 2017. Scavengers that fit beneath a microscope lens. Ecology 98:2725–2726. doi:10.1002/ecy.1874 28605015

[85] Radja A, Horsley EM, Lavrentovich MO, Sweeney AM. 2019. Pollen cell wall patterns form from modulated phases. Cell 176:856–868. doi:10.1016/j.cell.2019.01.014 30735635

[86] Bosch M, Hepler PK. 2005. Pectin methylesterases and pectin dynamics in pollen tubes. Plant Cell 17:3219–3226. doi:10.1105/tpc.105.037473 16322606 PMC1315365

[87] Engel P, Martinson VG, Moran NA. 2012. Functional diversity within the simple gut microbiota of the honey bee. Proc Natl Acad Sci U S A 109:11002–11007. doi:10.1073/pnas.1202970109 22711827 PMC3390884

[88] Love RR, Weisenfeld NI, Jaffe DB, Besansky NJ, Neafsey DE. 2016. Evaluation of DISCOVAR de novo using a mosquito sample for cost-effective short-read genome assembly. BMC Genomics 17:187. doi:10.1186/s12864-016-2531-7 26944054 PMC4779211

[89] Parks DH, Imelfort M, Skennerton CT, Hugenholtz P, Tyson GW. 2015. CheckM: assessing the quality of microbial genomes recovered from isolates, single cells, and metagenomes. Genome Res 25:1043–1055. doi:10.1101/gr.186072.114 25977477 PMC4484387

[90] Overbeek R, Olson R, Pusch GD, Olsen GJ, Davis JJ, Disz T, Edwards RA, Gerdes S, Parrello B, Shukla M, Vonstein V, Wattam AR, Xia F, Stevens R. 2014. The SEED and the rapid annotation of microbial genomes using subsystems technology (RAST). Nucleic Acids Res 42:D206–14. doi:10.1093/nar/gkt1226 24293654 PMC3965101

[91] Segata N, Börnigen D, Morgan XC, Huttenhower C. 2013. PhyloPhlAn is a new method for improved phylogenetic and taxonomic placement of microbes. Nat Commun 4:2304. doi:10.1038/ncomms3304 23942190 PMC3760377

[92] Katoh K, Standley DM. 2013. MAFFT multiple sequence alignment software version 7: improvements in performance and usability. Mol Biol Evol 30:772–780. doi:10.1093/molbev/mst010 23329690 PMC3603318

[93] Nguyen L-T, Schmidt HA, von Haeseler A, Minh BQ. 2015. IQ-TREE: a fast and effective stochastic algorithm for estimating maximum-likelihood phylogenies. Mol Biol Evol 32:268–274. doi:10.1093/molbev/msu300 25371430 PMC4271533

[94] Yang Z. 2007. PAML 4: phylogenetic analysis by maximum likelihood. Mol Biol Evol 24:1586–1591. doi:10.1093/molbev/msm088 17483113

[95] Csűös M. 2010. Count: evolutionary analysis of phylogenetic profiles with parsimony and likelihood. Bioinformatics 26:1910–1912. doi:10.1093/bioinformatics/btq315 20551134

[96] Syberg-Olsen MJ, Garber AI, Keeling PJ, McCutcheon JP, Husnik F. 2022. Pseudofinder: detection of pseudogenes in prokaryotic genomes. Mol Biol Evol 39:msac153. doi:10.1093/molbev/msac153 35801562 PMC9336565

[97] Bertelli C, Laird MR, Williams KP, Lau BY, Hoad G, Winsor GL, Brinkman FSL, Simon Fraser University Research Computing Group. 2017. IslandViewer 4: expanded prediction of genomic islands for larger-scale datasets. Nucleic Acids Res 45:W30–W35. doi:10.1093/nar/gkx343 28472413 PMC5570257

[98] Arndt D, Grant JR, Marcu A, Sajed T, Pon A, Liang Y, Wishart DS. 2016. PHASTER: a better, faster version of the PHAST phage search tool. Nucleic Acids Res 44:W16–W21. doi:10.1093/nar/gkw387 27141966 PMC4987931

[99] Kassambara A. 2020. Pipe-friendly framework for basic statistical tests [R package rstatix version 0.6.0]

[100] R Core Team. 2021. R: a language and environment for statistical computing. R Foundation for Statistical Computing, Vienna, Austria.

[101] Jumper J, Evans R, Pritzel A, Green T, Figurnov M, Ronneberger O, Tunyasuvunakool K, Bates R, Žídek A, Potapenko A, et al.. 2021. Highly accurate protein structure prediction with AlphaFold. Nature 596:583–589. doi:10.1038/s41586-021-03819-2 34265844 PMC8371605

[102] Mirdita M, Schütze K, Moriwaki Y, Heo L, Ovchinnikov S, Steinegger M. 2022. ColabFold: making protein folding accessible to all. Nat Methods 19:679–682. doi:10.1038/s41592-022-01488-1 35637307 PMC9184281

[103] Pettersen EF, Goddard TD, Huang CC, Meng EC, Couch GS, Croll TI, Morris JH, Ferrin TE. 2021. UCSF ChimeraX: structure visualization for researchers, educators, and developers. Protein Sci 30:70–82. doi:10.1002/pro.3943 32881101 PMC7737788

[104] The UniProt Consortium. 2017. UniProt: the universal protein knowledgebase. Nucleic Acids Res 45:D158–D169. doi:10.1093/nar/gkw1099 27899622 PMC5210571

