# Supplementary material for: Genome evolution following an ecological shift in nectar-dwelling Acinetobacter
Source: mSphere. 2024 Dec 26;10(1):e01010-24. doi: 10.1128/msphere.01010-24 (PMC11774029; doi:10.1128/msphere.01010-24)
Supplement: Supplemental information — Supplemental figures and tables. [file msphere.01010-24-s0001.docx]

**Supplementary Figures**

Fig. S1. Gene gains and losses within the nectar-dwelling tips. A subset of the phylogenomic tree from Fig. 1 is shown, with gains and losses at each tip given with a (+) or (-), respectively.

Fig. S2. Number of orthologs in CAZy functional categories from environmental and nectar-dwelling *Acinetobacter* genomes.


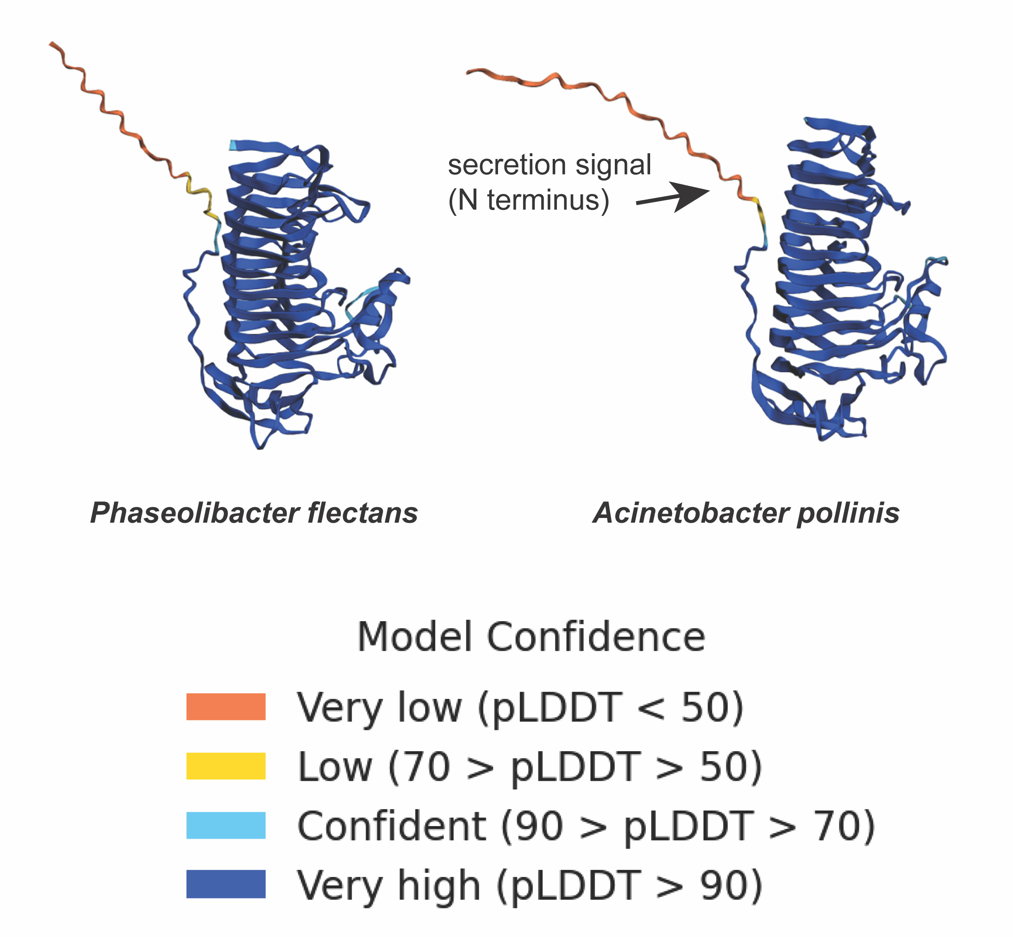


Fig. S3. Alphafold protein predictions showing confidence scores of polygalacturonase genes from the plant pathogen *Phaseolibacter flectens* (JAEE01000003.1) and *A. pollinis* FNA3 (locus_tag I2F29_RS02465). The pLDDT scores are a per-residue estimate of confidence on a scale from 1-100. Secretion signal domains at the N-terminus have low model confidence because they do not fold.

**Supplementary Tables**

Table S1: Genome sizes of *Acinetobacter* from GenBank. This includes all complete, reference genomes (one per species).

File attached separately: genome_sizes.xlsx

Table S2: All orthologs with RAST functional categories shown. Orthologs involved in nitrogen and amino acid metabolism and transport are given in a separate sheet.

File attached separately: orthologs_functional_categories.xlsx

Table S3. Orthologs in environmental, nectar, or all isolate genomes. Core orthologs are in all genomes, unique orthologs are only found in genomes of a given category.

|  | **Environmental isolates** | **Nectar Isolates** | **All isolates** |
| --- | --- | --- | --- |
| Total orthologs | 5,558 | 4,413 | 7,334 |
| Core orthologs | 1,377 | 2,720 | 1,076 |
| Unique to clade | 2,921 | 1,776 | NA |

Table S4: ANOVA results across functional categories, comparing ortholog numbers in environmental isolate genomes and nectar-dwelling isolate genomes. Analyses used untransformed ortholog numbers or values normalized by total ortholog number in each genome. We show per genome mean in each group and adjusted p values from Tukey’s HSD tests. Separate tabs for categories reduced in size and increased in size are given, where the results of the untransformed and normalized data are consistent. Within each tab, categories are order by the relative amount of change in nectar-dwelling genomes compared to environmental genomes. The “other” tab gives results for categories that were inconsistent depending on normalization. Only categories and subcategories with greater than five orthologs are included, and uncategorized orthologs were excluded.

File attached separately: ANOVA_functional_categories.xlsx

Table S5: Island viewer and Phaster results

| Strain | Genomic islands | Gene count on islands | Intact prophage |
| --- | --- | --- | --- |
| EC31 | 7 | 111 | 1 |
| EC34 | 7 | 123 | 1 |
| BB362 | 8 | 189 | 2 |
| EC115 | 11 | 210 | 0 |
| EC24 | 11 | 188 | 3 |
| BB226 | 12 | 256 | 0 |
| B5B | 13 | 326 | 2 |
| B10A | 14 | 352 | 0 |
| FNA11 | 14 | 286 | 0 |
| FNA3 | 15 | 248 | 0 |
| SCC474 | 15 | 310 | 3 |
| SCC477 | 15 | 332 | 3 |

Table S6: Copies of pectin degrading genes within the nectar-dwelling *Acinetobacter* clade and outgroup loci. GenBank accession numbers for all loci in nectar-dwelling *Acinetobacter* genomes are given, as are those for loci from plant pathogens used in phylogenetic analysis (Fig. 3). Isolates that contained at least one copy of pectin degrading genes in genomic islands are marked with an asterisk.

| **Isolate** | **Genome** | **Pectin lyase** | **Loci** | **Polygalac-turonase** | **Loci** |
| --- | --- | --- | --- | --- | --- |
| *Nectar-dwelling* Acinetobacter | | | | | |
| *A. pollinis* | |  |  |  |  |
| SCC474 | NZ_VTDR01000090 | ***3** | I2F30_RS13600  I2F30_RS12835  I2F30_RS13360 | **5** | I2F30_RS13595 I2F30_RS12730 I2F30_RS12855 I2F30_RS13355  I2F30_RS03310 |
| SCC477 | VTDQ01000043 | ***3** | I2F23_RS13535  I2F23_RS12130  I2F23_RS12035 | ***6** | I2F23_RS12030 I2F23_RS12120 I2F23_RS13540 I2F23_RS0208 I2F23_RS12040 I2F23_RS02080 |
| FNA11 | NZ_VTDS01000027 | **2** | I2F38_RS11510  I2F38_RS13050 | ***5** | I2F38_RS11505 I2F38_RS12850 I2F38_RS13055 I2F38_RS04145 |
| FNA3 | NZ_VTDT01000031 | ***2** | I2F29_RS11085  I2F29_RS12920 | ***5** | I2F29_RS11025 I2F29_RS12745 I2F29_RS12925 I2F29_RS02465 |
| *A. nectaris* | |  |  |  |  |
| CIP 110549 | AYER01000007 | **1** | AYER01000003 | **1** | P256_01834 |
| EC031 | JAERJC010000004 | **1** | JK152_03155 | **1** | JK152_04600 |
| EC034 | JAERJB010000015 | **1** | JK153_00405 | **1** | JK153_07735 |
| BB226 | JAEQDM010000003 | **1** | JKI99_01090 | **1** | JKI99_01610 |
| BB362 | JAEQDL010000011 | **1** | JKI98_04120 | **1** | JKI98_05235 |
| *A. rathckeae* | |  |  |  |  |
| EC024 | VTDO01000012 | **0** |  | **1** | I2F31_12745 |
| EC115 | VTDO01000012 | **0** |  | **1** | I2F31_12745 |
| *A. baretiae* | |  |  |  |  |
| B10A | NZ_VTDM01000023 | **0** |  | **1** | I2F17_RS09700 |
| B5B | NZ_VTDL01000029 | **0** |  | **1** | I2F27_RS10945 |
| *A. boissieri* ANC 4422 | NZ_FMYL01000001 | **0** |  | **2** | BLS38_RS01625 BLS38_RS12295 |
| *A. apis* ANC 5114 | NZ_FZLN01000001 | **1** | CFY84_RS07245 | **1** | CFY84_RS01715 |
| *Plant pathogens (not* Acinetobacter*)* | | | | | |
| *Erwinia pyrifoliae* | VOIC01000002 |  |  |  | FQ626_10560 |
| *Pectobacterium carotovorum* | NC_012917 |  |  |  | PC1_RS04935 |
| *Phaseolibacter flectans* | JAEE01000000 |  | JAEE01000014.1 |  | JAEE01000003.1 |
| *Pectobacterium odoriferum* | NZ_MTAN01000008 |  | BV923_RS11095 |  |  |
| *Pectobacterium wasabiae* | NZ_JQOH01000006 |  | KU73_RS13600 |  |  |
| *Dickeya zeae* | AJVN01000013 |  | AJVN01000013.1 |  |  |
| *Pseudomonas fluorescens* | JAIOKW010000018 |  | K7431_18850 |  |  |

Table S7: PAML amino acid selection test results from polygalacturonase and pectin lyase orthologs in nectar-dwelling *Acinetobacter* and outgroups (Table S6).

File attached separately: selection_pectin_genes.xlsx
